# Lifestyle variables and the risk of myocardial infarction in the General Practice Research Database

**DOI:** 10.1186/1471-2261-7-38

**Published:** 2007-12-18

**Authors:** Joseph AC Delaney, Stella S Daskalopoulou, James M Brophy, Russell J Steele, Lucie Opatrny, Samy Suissa

**Affiliations:** 1 Department of Epidemiology, Biostatistics and Occupational Health, McGill University, Montreal, Canada; 2 Division of Clinical Epidemiology, McGill University Health Center, Montreal, Canada; 3 Division of Cardiology, Royal Victoria Hospital/McGill University Health Center, Montreal, Quebec, Canada; 4 Department of Mathematics and Statistics, McGill University, Montreal, Canada; 5 Division of Internal Medicine, McGill University Health Center, Montreal, Canada

## Abstract

**Background:**

The primary objective of this study is to estimate the association between body mass index (BMI) and the risk of first acute myocardial infarction (AMI). As a secondary objective, we considered the association between other lifestyle variables, smoking and heavy alcohol use, and AMI risk.

**Methods:**

This study was conducted in the general practice research database (GPRD) which is a database based on general practitioner records and is a representative sample of the United Kingdom population. We matched cases of first AMI as identified by diagnostic codes with up to 10 controls between January 1^st^, 2001 and December 31^st^, 2005 using incidence density sampling. We used multiple imputation to account for missing data.

**Results:**

We identified 19,353 cases of first AMI which were matched on index date, GPRD practice and age to 192,821 controls. There was a modest amount of missing data in the database, and the patients with missing data had different risks than those with recorded values. We adjusted our analysis for each lifestyle variable jointly and also for age, sex, and number of hospitalizations in the past year. Although a record of underweight (BMI <18.0 kg/m^2^) did not alter the risk for AMI (adjusted odds ratio (OR): 1.00; 95% confidence interval (CI): 0.87–1.11) when compared with normal BMI (18.0–24.9 kg/m^2^), obesity (BMI ≥30 kg/m^2^) predicted an increased risk (adjusted OR: 1.41; 95% CI: 1.35–1.47). A history of smoking also predicted an increased risk of AMI (adjusted OR: 1.81; 95% CI: 1.75–1.87) as did heavy alcohol use (adjusted OR: 1.15; 95% CI: 1.06–1.26).

**Conclusion:**

This study illustrates that obesity, smoking and heavy alcohol use, as recorded during routine care by a general practitioner, are important predictors of an increased risk of a first AMI. In contrast, low BMI does not increase the risk of a first AMI.

## Background

Obesity is a growing public health problem that is associated with an increased rate of cardiovascular events. About one in three patients admitted to hospital with acute coronary syndrome in Europe were obese with additionally half of the patient population being overweight [1].

Clinical databases based on general practice records are a potentially useful source of information (when it is available) for studying the magnitude of risk factors such as obesity, smoking and heavy alcohol use at the population level in a real-world setting. However, these databases often have missing data on some patients which needs to be properly accounted for in any analysis. Several methods exist [2,3], but multiple imputation has been systematically shown to be superior to case deletion and indicator variable methods in reducing bias [4-6].

As obesity is a growing public health concern, it is important to identify the impact of body mass index (BMI) in the occurrence of the first acute myocardial infarction (AMI). The primary objective of this study is to estimate the association between BMI and the risk of the first AMI. As a secondary objective, we considered the association between other lifestyle variables, smoking and heavy alcohol use, and AMI risk. Finally, we sought to determine if the choice of how to deal with missing information was important.

## Methods

### Study population

This study is based on the United Kingdom's General Practice Research Database (GPRD) [7]. This is a large clinical database based on the medical charts of general practitioners. It records information such as prescriptions issued and medical diagnoses made using the United Kingdom specific READ and OXMIS medical codes. The recorded information on drug exposure and diagnoses has been validated and proven to be of high quality [7,8]. The GPRD also records information on factors such as BMI, blood pressure, smoking and alcohol consumption [7]. However, these variables are reported by validation studies to have non-trivial amounts of missing data [7,8] and this can lead to biased estimates of effect [9].

We identified all first-ever AMIs recorded in the GPRD between January 1^st^, 2001 and December 31^st^, 2005 using the medical codes recorded in the database as our cases. These medical codes are described in Additional File 1. To be eligible to be selected as a case, a patient needed to be at least 18 years of age and have no previous record of an AMI before the index event. The date recorded in the database for the first AMI was taken as the index date for the case. We matched each case with up to 10 controls based on age (± 2 years), GPRD practice and index date. On the index date, the control must not have had a previous AMI, must still be registered in the GPRD and be alive to be eligible as a control.

Both cases and controls were required to have at least 3 years of follow-up in the GPRD before the index date to allow adequate time to assess comorbid conditions.

BMI was defined as the most recently available pre-AMI body weight (in kilograms) divided by the square of the height (in meters) (kg/m^2^) and was used to categorize patients according to the World Health Organization's definition [10]: underweight (BMI: <18.0 kg/m^2^), normal weight (BMI: 18.0–24.9 kg/m^2^), overweight (BMI: 25–29.9 kg/m^2^) and obese (BMI: ≥30 kg/m^2^).

For smoking we grouped subjects into the categories of never smokers and ever smokers. For heavy alcohol use we used at least one clinical diagnosis recorded in the database. For BMI and smoking status, we used the closer to the index date recorded value in the database. However, for most patients BMI and smoking status are recorded only once in the GPRD [7].

Ethical review for this study was done by the Independent Scientific Advisory Committee for MHRA database research

### Statistical Analysis

Conditional logistic regression was used to estimate the odds ratios (ORs) for the different BMI categories. We handled missing data using three different typical approaches (case deletion, indicator variable and multiple imputation). It was important to include a broad spectrum of covariates as predictors in our multiple imputation model [11,12]. We considered a crude model for BMI, smoking and heavy alcohol use, separately. Because of the cross-sectional nature of our data, we could not assess whether comorbidities preceded obesity, and so we did not adjust for these variables in our statistical models (although they were used in the multiple imputation to infer BMI). Instead, we limited our statistical adjustment to each lifestyle variable jointly, as well as age, sex and number of hospitalizations in the past year (as a proxy for overall health status).

More details of the imputation and analysis are discussed in Additional File 2.

## Results

We identified 19,353 cases of AMI which were matched to 192,821 controls. Selected characteristics of the cases and the controls are described in Table 1. The pattern of missing data in this study is also shown in Table 1 as are the post-imputation results of some variables. The cases have higher rates and levels of known cardiovascular risk factors including diabetes and angina as well as elevated blood pressure and serum cholesterol levels.

**Table 1:** Lifestyle information and percentage of missing data in subjects comparing patients acute myocardial infarction (cases) to the general population from which cases arose (controls).

**Basic Descriptive Statistics**	**Cases (n = 19,353)**	**Controls (n = 192,821)**
Mean age (SD)	70.0 (13.1)	69.9 (13.0)
Male	54.4%	44.1%
% heavy alcohol use	3.6%	2.3%
# hospitalizations/past year (SD)	0.33 (1.14)	0.16 (0.81)
**Variables**	**Rates of missing values (%)**	
*Smoking*	8.4	12.3
*Body Mass Index*	20.6	23.5
*Blood Pressure*	30.6	43.6
*Estimated Systolic Blood Pressure (SD)*	144.1 (18.7)	142.2 (17.7)
*Estimated Diastolic Blood Pressure (SD)*	81.0 (10.1)	80.4 (9.0)
*Serum Cholesterol*	64.4	76.2
*Estimated Serum Cholesterol (SD)*	5.54 (1.16)	5.49 (1.20)
**Key Comorbidities**		
*Diabetes*	15.8%	9.1%
*Angina*	20.2%	11.8%
*Renal Failure*	3.0%	1.1%
*Chronic Obstructive Pulmonary Disease*	8.2%	5.1%
*Stroke*	5.5%	3.5%

* SD: standard deviation.

Table 2 describes the distribution of BMI and smoking among subjects with imputed values for BMI as opposed to subjects with measured BMI. Of note is that the size of the underweight category is much greater among those subjects with imputed BMIs; it is 1.8% versus 3.8% in the cases and 1.8% versus 4.7% in the controls. In general, patients with imputed values have systematically lower rates of smoking and lower BMI values than subjects with recorded information.

**Table 2:** Comparison of distributions of body mass index and smoking among subjects with measured body mass index values and those with imputed body mass index values.

	**Cases**		**Controls**	
	**Measured (N = 15,423)**	**Imputed (N = 3,930)**	**Measured (N = 146,725)**	**Imputed (N = 46,096)**

**Body Mass Index**
<18.0	1.8%	3.8%	1.7%	4.7%
18.0–24.9	35.2%	35.6%	40.9%	39.9%
25.0–29.9	40.8%	40.7%	39.0%	38.6%
≥30.0	22.1%	19.9%	18.3%	16.8%
Mean (SD)	26.8 (4.8)	26.2 (4.6)	26.3 (4.7)	25.6 (4.6)
**Smoking**
Ever	56.9%	53.6%	40.9%	40.0%
Never	43.1%	46.7%	59.0%	60.0%

SD: standard deviation.

Table 3 describes the relationship between BMI and the rate of AMI. A pronounced increased risk in the obese patients was found regardless of how we account for missing data. Using the adjusted estimates from the multiple imputation analysis, there is an increase in risk in the obese (adjusted OR: 1.41; 95% confidence interval (CI): 1.35–1.47). The change in adjusted OR for the underweight, as based on different methods of handling missing data, was the most important with a 15.3% change in the estimate between case deletion (adjusted OR: 1.15; 95% CI: 0.96–1.37) and multiple imputation (adjusted OR: 1.00; 95% CI: 0.87–1.11).

**Table 3:** Relationship between body mass index and acute myocardial infarction using three different methods to account for missing values (odds ratio, 95% confidence interval). The normal BMI category (18.0–24.9 kg/m^2^) was used as the reference group.

**Body mass index **(kg/m^2^)	**Case Deletion**	**Indicator Variable**	**Multiple Imputation (10 copies)**
**Crude Estimates of Effect**
<18.0	1.23 (1.03–1.46)	1.21 (1.02–1.44)	1.03 (0.91–1.17)
18.0–24.9	Reference	Reference	Reference
25.0–29.9	1.21 (1.15–1.27)	1.21 (1.15–1.27)	1.20 (1.16–1.24)
≥30.0	1.35 (1.27–1.43)	1.35 (1.27–1.43)	1.40 (1.34–1.46)
Missing Indicator	n/a	0.96 (0.91–1.02)	n/a
Adjusted* Estimates of Effect
<18.0	1.15 (0.96–1.37)	1.13 (0.95–1.35)	1.00 (0.87–1.11)
18.0–24.9	Reference	Reference	Reference
25.0–29.9	1.18 (1.12–1.24)	1.18 (1.12–1.24)	1.16 (1.14–1.21)
≥30.0	1.35 (1.27–1.44)	1.35 (1.27–1.44)	1.41 (1.35–1.47)
Missing Indicator	n/a	1.13 (1.06–1.20)	n/a

n/a: not applicable.

* matched for age, GPRD practice and index date and adjusted for age, sex, heavy alcohol use, smoking and number of hospitalizations in the past year.

Table 4 describes the results for ever smoker versus never smoker using the three different approaches for missing values. In this population sample we confirm the well-known finding that ever smoking is a strong risk factor for having an AMI (adjusted OR: 1.81; 95% CI: 1.75–1.87). This effect was consistently shown with all three different methods used to account for missing values.

**Table 4:** Relationship between smoking status and acute myocardial infarction as shown using three different methods to account for missing values and analyzed using conditional logistic regression (odds ratio, 95% confidence interval). The never smoking group was used as the reference.

**Smoking status**	**Case Deletion**	**Indicator Variable**	**Multiple Imputation (10 copies)**
**Crude Estimates of Effect**
Ever	1.92 (1.84–2.00)	1.92 (1.84–2.00)	1.90 (1.84–1.97)
Never	Reference	Reference	Reference
Missing	n/a	0.88 (0.82–0.95)	n/a
Adjusted* Estimates of Effect
Ever	1.83 (1.75–1.91)	1.83 (1.75–1.91)	1.81 (1.75–1.87)
Never	Reference	Reference	Reference
Missing	n/a	0.86 (0.79–0.94)	n/a

n/a: not applicable.

* matched for age, GPRD practice and index date and adjusted for age, sex, heavy alcohol use, smoking and number of hospitalizations in the past year.

Furthermore, subjects with a clinical diagnosis of heavy alcohol use appeared to have a small increased risk of a first AMI (adjusted OR: 1.15; 95% CI: 1.06–1.26).

## Discussion

This is the first study evaluating the association between BMI and the first AMI, using a clinical database based on general practitioner records (GPRD). It is a case-control study that includes a large sample of consecutive, unselected cases with AMI and matched controls. Therefore, it reflects real life data including a large proportion of female and elderly patients. In this study we also assessed the impact of smoking and heavy alcohol use on the occurrence of the first AMI. We used three different methods to account for missing data, namely case deletion, indicator variable and the more sophisticated multiple imputation method.

### BMI as a risk factor

In our study, we observe that low and normal BMI values are not associated with an increased risk of a first AMI but that high BMI values are. This shape could be described as a J-shaped association between BMI categories in which we have no effect on one direction from normal and an increased risk in the other. To our knowledge, this is the first study to describe this effect for first AMI in a United Kingdom population sample.

Despite previous research, controversy remains regarding the relationship between BMI and AMI [13-16]. Some studies have shown that BMI has a U-shaped effect (bimodal occurrence) of adverse events and adverse outcomes with an increased risk in underweight and morbidly obese people, but with a lower risk for overweight and obese when compared to normal-weight patients. However, these studies have often not comprehensively accounted for potential sources of confounding, with underestimates of the effect of overweight and obesity on longevity and overestimates of the risks of leanness. Major potential sources of bias particular to studies of BMI and mortality include (1) failure to adequately account for missing values, (2) failure to adequately account for potential sources of confounding (e.g. pre-existing disease or concomitant illnesses such as cancer, leukemia and lymphoma), (3) unmeasured factors that affected outcomes, and (4) inappropriate adjustment for the biological effects of obesity (i.e. for conditions that included in the causal pathway between obesity and AMI), including hypertension and diabetes. Also some prior studies are not very informative as they are hospital-based and they focus on the outcome after AMI. Furthermore, a study of AMI patients followed for 8–10 years showed that although overall obesity (as assessed by BMI) is inversely related to mortality, abdominal obesity appears to be an independent predictor of all-cause mortality in men and perhaps also in women [17].

Other studies have found similar results with ours but mostly for mortality. In the Multifactor Primary Prevention Study, when the BMI category 20.0–22.5 kg/m^2 ^was used as the reference group, the underweight group did not carry a higher risk for an AMI (adjusted Hazard Ratio (HR): 1.08; 95% CI: 0.76–1.52) or for coronary artery bypass graft without prior AMI (adjusted HR: 0.86; 95% CI: 0.25–2.90). However, overweight and obese patients were carrying a higher risk for AMI when compared with the normal BMI category [18].

In a prospective study of more than 1,000,000 adults in the United States the curve for the risk of death from cardiovascular disease among subjects who never smoked and had no history of disease was J-shaped; this indicated that a high BMI was most predictive of death from cardiovascular disease than a low BMI. However, the curve for the risk of death from all other causes was U-shaped [19].

A recent meta-analysis including 302,296 participants worldwide and 18,000 coronary heart disease events during follow-up showed that there was an increased risk for coronary events associated with overweight and obesity; the adjusted relative risk (and 95% CI) was 1.32 (1.24–1.40) for BMI of 25.0 to 29.9 kg/m^2 ^and 1.81 (1.56–2.10) for BMI ≥30 kg/m^2^, when compared with normal BMI [20].

### Smoking and heavy alcohol use as risk factors

An increased risk of a first AMI was associated with ever smoking. This finding was consistently found regardless of the method used to deal with missing values. This strong association between smoking and AMI has been shown before. For example, the INTERHEART study found that tobacco use is one of the most important causes of AMI globally, especially in men. The risk for AMI was increased regardless of the form of tobacco use, including different types of smoking and chewing tobacco and inhalation of second hand tobacco smoke [21].

Another study also found that the type or yield of cigarettes did not result in significantly different findings, with similar risk for smoker of low versus high tar cigarettes [22].

Heavy alcohol use was also consistently associated with a higher risk of a first AMI in our study. Heavy alcohol use is a known risk factor for cardiovascular risk. The INTERHEART study, among others, also found this association [23].

### Missing data

We used three common methods to deal with missing data. In cases where there is a difference between the results of the case deletion, indicator variable and multiple imputation, simulation studies have demonstrated the superiority of multiple imputation method when missing data exceed 10% of the total [6]. In our study, only smoking met that criterion <10% missing among cases and only slightly more among controls. In all methods, smoking was a strong risk factor for AMI, with little to no change in estimate as we accounted for missing data with different methods.

The pattern of obesity by measured versus unmeasured BMI (as shown in Table 2) demonstrates the circumstances under which multiple imputation will make a difference in the results of a study. The only category of weight shows important differences in the estimates of the effect of BMI on AMI between those with a measure of BMI and those without one is the underweight. In the underweight we found a 15.3% difference in the estimates of the risk of AMI between using case deletion versus multiple imputations to handle missing data. While we are fortunate in this case not to have this bias shift the inference (as neither is statistically significant), this is not guaranteed in future studies. In such cases, the estimate from multiple imputation should be preferred [4-6,12].

### Strengths and limitations

This is a broad and unselected population sample of the United Kingdom population that allows us to infer the current levels of risk. Due to the comprehensive nature of the covariates in the database, we were able to use an extremely rich wealth of information in imputing missing data. This allows us to describe the empirical risk associated with different levels of BMI as seen by general practitioners. Recently, the INTERHEART study also reported that, among others, smoking, obesity, bad dietary habits and alcohol intake, as well as lack of regular physical activity account for most of the risk of AMI worldwide in both sexes and at all ages in all regions [23].

However, this study also has several limitations. We defined the "first AMI" as the first event occurred after at least 3 consecutive years of being followed in the GPRD and being free of an AMI. This might also include some patients who had their AMI after long intervals. However, as there is very good validation for hospital referrals (and communication with specialists) [7], it is very uncommon if a patient with a previous MI was not followed by a Cardiologist/Specialist and/or did not have any follow-up tests for at least 3 years. Also in general, in database studies collection of data is often less standardized or less accurate; however, GPRD is a popular database and many validation studies have proven satisfactory accuracy and completeness of the data [7]. Despite adjustments using multivariate analyses, unmeasured factors that affected outcomes were likely present. The BMI was used as a marker for total body fat, while the distribution of body fat is unknown. However, there is evidence supporting that there is a good correlation between BMI and central obesity, a known risk factor for cardiovascular events [24]. We treated smoking as a binary variable. This approach has been known to be subject to misclassification [25] in the GPRD. However, we avoided classifying the patients as never, current and ex smokers as the GPRD does not systematically track quitting and starting patterns among smokers. Also there is no information on the duration, intensity or type of smoking available in the GPRD. The same limitation applies for the clinical diagnosis of heavy alcohol use. We do not have information on the severity of AMI; it was shown that different levels of healthy lifestyle are associated with the severity of cardiac events and outcomes after the event [26].

Furthermore, we are not able to verify the assumption that the missing data were ignorable (an assumption of multiple imputation in that the missing data can be completely predicted from the observed data) [4-6,12]. It is possible that more information would be required to generate an unbiased prediction of the data than is present in this database and this cannot be tested without this data. However, it is quite plausible that the nature of data collection in the GPRD will be such that the data is not missing at random and so the estimates of missing BMI values should be interpreted with caution.

**Figure 1:**
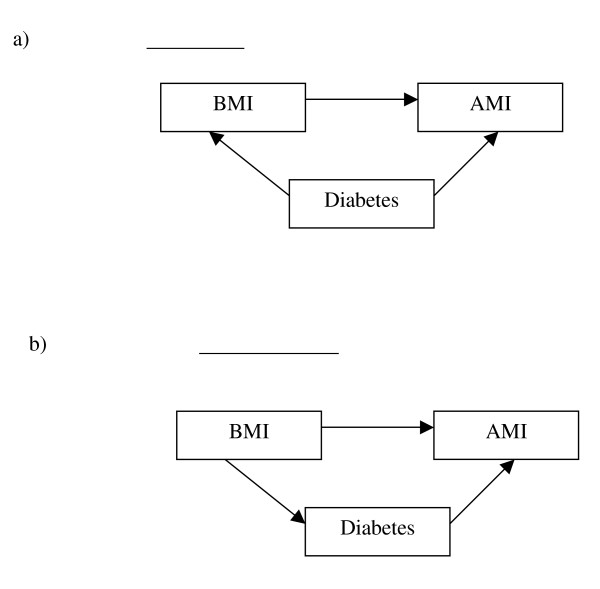
Directed Acyclic Graphs (DAGs) showing the difference between a) a confounding variable and b) a variable on the causal pathway. Here the example of body mass index (BMI) (exposure), acute myocardial infarction (AMI) (outcome) and diabetes (covariate) is used. The statistical approaches in this paper assume case b) for the comorbid conditions listed in table 1.

The estimates of effect found in this paper are not protected against misclassification of the exposure. Also, the temporal sequence of variables that are measured cross-sectionally (like BMI) in the GPRD cannot be captured. As can be seen in Figure 1, the analysis of these variables requires assumptions about whether the covariate is a common cause of the exposure and the outcome (and thus a confounder) [27,28] or if it lies in the causal pathway between the exposure and the outcome (and should not be adjusted for). Our study makes the assumption, as has been seen in other contexts [27], that the estimate adjusted only for age and sex is the correct model given our understanding of the relationships between the candidate confounders and the exposure. Future researchers, however, can and should test these conceptual models with longitudinal data.

## Conclusion

Future work in this area in the GPRD should account for the properties of the missing data in this database. However, once the missing data are properly accounted for, the GPRD appears to be a rich source of data on lifestyle risk factors at the population level. The interesting finding of a J-shaped relationship between BMI and risk of first AMI, while seen for mortality in previous work, is novel for first AMI and should be explored further.

This work on obesity can be extended to other areas where the relationship between obesity and the disease is less well-known [29]. Meanwhile, physicians should continue to advise patients to try and modify lifestyle factors, where possible, to reduce AMI risk.

## Competing interests

The author(s) declare that they have no competing interests.

## Authors' contributions

All authors contributed to the conception and design of the study. JD and RS conducted the statistical analysis of the paper. All authors contributed to the interpretation of the data and developed the statistical analysis. JD and SD wrote the paper with critical contributions from all authors. The final manuscript was approved by all authors.

## Pre-publication history

The pre-publication history for this paper can be accessed here:



## Supplementary Material

Additional file 1 List of medical codes used to identify the first acute myocardial infarction. This file documents the READ and OXMIS medical codes that were used to identify the event of myocardial infarction in this study. Click here for file

Additional file 2 Imputation variables and method. This file contains more detailed information about the statistical methods used to implement multiple imputation to handle missing data in the paper. Click here for file
