# Supplementary material for: Lifestyle variables and the risk of myocardial infarction in the General Practice Research Database
Source: BMC Cardiovasc Disord. 2007 Dec 18;7:38. doi: 10.1186/1471-2261-7-38 (PMC2241637; doi:10.1186/1471-2261-7-38)
Supplement: Additional file 1 — List of medical codes used to identify the first acute myocardial infarction. This file documents the READ and OXMIS medical codes that were used to identify the event of myocardial infarction in this study. [file 1471-2261-7-38-S1.doc]

**Additional File 1: List of medical codes used to identify the first acute myocardial infarction**

| **Read / OXMIS Code** | **Term Type** | **Read / OXMIS Term** |
| --- | --- | --- |
| 323..00 | READ | ECG: myocardial infarction |
| G30X.00 | READ | Acute transmural myocardial infarction of unspecified site |
| G361.00 | READ | Atrial septal defect/current comp follow acute myocardial infarction |
| G361.00 | READ | Atrial septal defect/current comp follow acute myocardal infarction |
| G362.00 | READ | Ventric septal defect/current comp follow acute myocardial infarction |
| G362.00 | READ | Ventric septal defect/current comp follow acute myocardial infarction |
| 4100N | OXMIS | Myocardial infarction with hypertension |
| 4109TE | (OXMIS) | Thrombosis endocardial |
| 4109TM | OXMIS | Myocardial thrombosis |
| 4119N | (OXMIS) | Subendocardial infarction |
| 14A4.00 | READ | H/O: myocardial infarct >60 |
| 3234 | READ | ECG: posterior/inferior infarction |
| G304.00 | READ | Posterior myocardial infarction NOS |
| G308.00 | READ | Inferior myocardial infarction NOS |
| G30y200 | READ | Acute septal infarction |
| G366.00 | READ | Thrombosis atrium |
| G366.00 | READ | Thrombosis atrium |
| 4129MC | (OXMIS) | Sclerosis myocardial |
| 4140 | OXMIS | Asymptomatic hypertensive ischaemic hear |
| G307.00 | READ | Acute subendocardial infarction |
| G34y100 | READ | Chronic myocardial ischaemia |
| G360.00 | READ | Haemopericardium/current comp follow acute myocardial infarction |
| G360.00 | READ | Haemopericardium/current comp follow acute myocardial infarction |
| G305.00 | READ | Lateral myocardial infarction NOS |
| 4109CR | (OXMIS) | Accident coronary |
| 4129N | (OXMIS) | Ischaemic heart disease asymptomatic |
| G30..15 | READ | MI - acute myocardial infarction |
| G300.00 | READ | Acute anterolateral infarction |
| G344.00 | READ | Silent myocardial ischaemia |
| G38..00 | READ | Postoperative myocardial infarction |
| 4129RE | (OXMIS) | Sclerosis endocardial |
| G302.00 | READ | Acute inferolateral infarction |
| G303.00 | READ | Acute inferoposterior infarction |
| 4109TL | (OXMIS) | Thrombosis mural |
| 3235 | READ | ECG: subendocardial infarction |
| G301.00 | READ | Other specified anterior myocardial infarction |
| G301000 | READ | Acute anteroapical infarction |
| G31y200 | READ | Subendocardial ischaemia |
| G5y1.00 | READ | Myocardial degeneration |
| 322..00 | READ | ECG: myocardial ischaemia |
| 322Z.00 | READ | ECG: myocardial ischaemia NOS |
| G30..17 | READ | Silent myocardial infarction |
| 4149 | (OXMIS) | Asymptomatic ischaemic heart disease |
| 14A3.00 | READ | H/O: myocardial infarct <60 |
| G381.00 | READ | Postoperative transmural myocardial infarction inferior wall |
| G306.00 | READ | True posterior myocardial infarction |
| G30..00 | READ | Acute myocardial infarction |
| G30z.00 | READ | Acute myocardial infarction NOS |
| G32..12 | READ | Personal history of myocardial infarction |
| G350.00 | READ | Subsequent myocardial infarction of anterior wall |
| 4100NA | OXMIS | Myocardial infarct acute with hypertension |
| 4109CL | OXMIS | Occlusion coronary |
| 4109N | OXMIS | Myocardial infarction |
| 4109NA | OXMIS | Myocardial infarction acute |
| 4109NH | OXMIS | Infarct heart |
| 4129AM | OXMIS | Myocardial ischaemia |
| 4109NC | (OXMIS) | Coronary infarction |
| 4129NS | (OXMIS) | Silent ischaemia |
