# Supplementary material for: Lifestyle variables and the risk of myocardial infarction in the General Practice Research Database
Source: BMC Cardiovasc Disord. 2007 Dec 18;7:38. doi: 10.1186/1471-2261-7-38 (PMC2241637; doi:10.1186/1471-2261-7-38)
Supplement: Additional file 2 — Imputation variables and method. This file contains more detailed information about the statistical methods used to implement multiple imputation to handle missing data in the paper. [file 1471-2261-7-38-S2.doc]

**Additional File 2: Imputation variables and method**

Age, male sex, the number of hospitalizations in the year before the index date and a history of heavy alcohol use were variables included in our multiple imputation; blood pressure readings and total cholesterol values were also included as these may be important markers of elevated BMI. Furthermore, a comprehensive list of comorbidities based on GPRD medical codes were used to look for possible markers of obesity- or smoking-related disease. This list included: diabetes mellitus, dyslipidemia, angina, congestive heart failure, cardiac arrthymia, stroke, peripheral arterial disease, chronic obstructive pulmonary disease, asthma, liver failure, renal failure, pulmonary embolism, deep vein thrombosis, peptic ulcer, gastrointestinal bleeding, arthritis, cancer (any), and dementia. These conditions needed to have been recorded on the patient’s electronic chart before the index date for the AMI. We also considered drug prescriptions in the 90 days before the index date for the following drug classes: oral antidiabetic agents, insulin, β-blockers, calcium channel blockers, diuretics, angiotensin-converting enzyme inhibitors, angiotensin receptor blockers, α-blockers, digoxin, statins, fibrates, aspirin, clopidogrel, non-steroidal anti-inflammatory agents, corticosteroids, gastric acid suppressants, antibiotics, and antidepressants.

It was important to include a broad spectrum of covariates as predictors in our multiple imputation model. Based on previous studies, we also included the outcome as a variable in the multiple imputation [11]. Furthermore, we included in the multiple imputation model medical codes recorded by general practitioners for clinical obesity, underweight and hypertension as supporting evidence for assessing the BMI of the patients. We also imputed cases and controls using separate multiple imputation steps.

All analyses were performed using SAS Software Version 9.1.3 (SAS Institute, Cary, NC, USA). We analyzed the data using case deletion, indicator variables and multiple imputations as different approaches to handling patients with missing observations in order to compare the differences between these methods. The multiple imputation of missing values was performed (using the function PROC MI) with all variables in the covariate section used to produce the values for imputation. We used mixed chain imputation with 1000 burn in iterations and the MCMC option. We assessed time-plots and auto-regression plots to determine that the imputation was well behaved and that there was minimal auto-regression between iterations of the MCMC algorithm. The results for each imputation were generated using conditional logistic regression (PROC PHREG) and then combined using PROC MIANALYZE. We used 10 imputed datasets for this study to ensure that our effect estimates were not overly inaccurate due to Monte Carlo variability [12].
